# Effect of emergency oral contraceptive use on condom utilization and sexual risk taking behaviours among university students, Northwest Ethiopia: a cross-sectional study

**DOI:** 10.1186/1756-0500-5-501

**Published:** 2012-09-13

**Authors:** Belaynew Wasie, Yeshambel Belyhun, Beyene Moges, Bemnet Amare

**Affiliations:** 1 Department of Epidemiology and Biostatistics, Institute of Public Health, University of Gondar, P.O. Box 196, Gondar, Ethiopia; 2 Department of Microbiology, Immunology and Parasitology, College of Medicine and Health Sciences, University of Gondar, Gondar, Ethiopia; 3 Department of Medical Biochemistry, College of Medicine and Health Sciences, University of Gondar, P.O. Box 196, Gondar, Ethiopia

**Keywords:** University students, Emergency contraceptive, Risky sexual behaviour, Condom use, Northwest Ethiopia

## Abstract

**Background:**

Young people between the ages of 15 and 24 years are both the most at risk of HIV and the greatest hope for turning the tide against HIV/AIDS. Although various surveys have been done on sexual behaviour of youth in Ethiopia, studies assessing the effect of emergency oral contraceptives on condom utilization of university students are lacking.

**Methods:**

A cross-sectional study was conducted in two major universities of Ethiopia from January to May 2011 using structured self administered questionnaire with the aim to assess the effect of introducing oral emergency contraceptive pills on condom utilization and sexual risk taking behaviours among female university students. Study participants were selected by simple random sampling using the list from the associate registrars of each University. Data were entered, cleaned and analyzed using SPSS version 17.0. Bivariate and multiple logistic regression analyses were used to determine factors associated with condom utilization.

**Results:**

a total of 623 students out of 660 were included giving response rate of 94.4%. A total of 103(16.5%) had history of sexual intercourse and nearly half (45.6%) of them had sex before the age of 20 years. Forty (6.4%) students had history of sexually transmitted infections (STI). Sixty seven percent of students had heard about emergency oral contraceptives. One hundred and ninety one (45.7%) of students believe that EOC is effective in preventing pregnancy. Believing that EOC is effective in preventing pregnancy (adjusted Odds ratio, AOR = 0.22 95% CI 0.06, 0.87), condom prevents STI (AOR = 10.37, 95% CI 1.73, 62.24) and younger age below 20 years (AOR = 11.68 95% CI 1.25, 109.19) were statistically significantly associated with condom use.

**Conclusion:**

a significant number of students had history of sexual intercourse and used emergency contraception. The belief in the effectiveness of EOC negatively affects condom use. The preference for the pill may make teenagers less prepared to practice STI protective behaviours in specific situations. Therefore, there is an urgent need to educate young people in universities about reproductive health and family planning and skills on how to prevent HIV/STIs including unwanted pregnancy.

## Background

For women at high risk for HIV acquisition and do not wish to become pregnant, contraceptive choice is a crucial issue. According to statistics reported by the United Nations Population Division, 49% of reproductive age women use modern contraceptive methods [1]. Oral contraceptives are the third most common contraceptive method used worldwide, and the most common modern method used in sub-Saharan Africa [1]. Emergency oral contraceptives are contraception administered after unprotected intercourse to prevent pregnancy. It is also known as "post-coital contraception", and is less effective than regular contraception. Emergency oral contraceptives (EOC) are intended for occasional or emergency use only and not as a regular contraception. It is associated with a failure rate of 0.2% to 3% [1,2].

Effective contraception has a key role to play in reducing maternal and infant mortality (by spacing pregnancies) and incidence of unwanted pregnancy. At the same time, the HIV/AIDS epidemic has reached crisis proportions in a number of sub-Saharan countries [2]. In Ethiopia, women account for a larger share of those directly affected by HIV/AIDS [3]. In 2006, the national HIV prevalence was estimated to have been 3% among males and 4% among females. In the same year, 55% of the estimated 1.32 million people living with HIV/AIDS (PLWHA) were women. They accounted for 54.5% of AIDS related deaths and 53.2% of new infections [4]. Despite the magnitude of the HIV/AIDS epidemic, the level of condom use among married or cohabiting couples in countries classified by UNAIDS as experiencing a generalized HIV epidemic remains very low [5].

Aside from abstinence, consistent condom use is the best protection against HIV and other sexually transmitted infections (STIs). Hormonal contraception methods such as birth control pills are clearly the best method for protection against pregnancy. Females who use the EOC to prevent pregnancy should also use a condom to protect against STI; however, research data indicate that females who use EOC do not necessarily consider using a condom for STI prevention [6]. In a large Canadian study, only 27% of current EOC users reported concurrent use of condoms [7].

In a cohort of commercial sex workers in Mombasa, Kenya with extremely high HIV-1 incidence, it was observed that women using EOC had an increased risk of HIV-1 acquisition compared with non-EOC users (OR = 4.5, 95% CI = 1.4–13.8) after adjustment for genital ulcer disease, Chlamidia infection and condom use [8]. Another study in Kenya reported that oral contraceptive pills (hazard ratio [HR], 1.5; 95% CI, 1.0–2.1) were associated with greater risk of HIV-1, compared with nonuser of contraception [9].

There is also an important element in policy debates over making EOC more widely available is a concern that it will increase risk taking. There is a concern that women who have easy access to a postcoital form of contraception may in fact have more unprotected intercourse and abandon more effective forms of regular contraception [10]. Studies have also shown that clinicians- as well as pharmacists-and users are concerned about the impact of increased access to EOC on sexual risk taking behaviours and STIs [11-13].

Research on the impact of EOC pills on condom utilization and sexual risk taking behaviours may help to inform policy-makers in Ethiopia. However, no research has been conducted in this area in the country. Therefore the aim of our study was to evaluate effect of introducing oral emergency contraceptive pills on condom utilization and sexual risk taking behaviours in female university students of Ethiopia. We hope that our study will provide baseline data to assist policy makers in developing appropriate evidence-based strategies to promote the use of condom in Ethiopia.

## Methods

### Study design and setting

A cross-sectional study was conducted in January at Gondar University but in April-May at Bahir Dar University which are 180 km apart each other. These universities are among oldest universities and each university enrols over 15000 students in Northwest Ethiopia, located 727 Km and 540 Km away from the capital city, Addis Ababa respectively. In Gondar University, this study was conducted in Tewodros campus, Maraki campus and College of Medicine and Health Sciences campus. Bahir Dar University has two campuses, however; this study was conducted only in one campus due to administrative reasons. According to the information obtained from the respective associate registrar offices, the total numbers of female students from the main campuses in 2011 were over 5900 and 4895 in Gondar University and Bahir Dar University, respectively.

### Sample size and sampling procedure

Sample size was determined using single population proportion formula with the assumptions: d = maximum allowable error (4%), P = proportion of students having awareness of emergency contraception (50%), Z statistic = 1.96 and non-response 10% giving a final sample size of 660 students. The students were selected by simple random sampling using the list of students in each faculty registrar and a table of random numbers was utilized for identification of the serial numbers of each student.

### Study variables

The outcome variable of the study was condom utilization while the explanatory variables included socio-demographic, sexual and reproductive health characters, knowledge of contraceptive methods, Attitude towards contraception, and utilization of EOCs.

### Data collection procedures

We used a five-page pre-tested and structured self administered questionnaire in English and translated to Amharic [National language] was used to collect data. The questionnaire had both closed and open-ended questions. It was composed of four parts. The first part, which was the preamble, contained the following definition of "Emergency contraceptive pills are also called morning-after pills. They are used to prevent pregnancies after unprotected sexual intercourse or after condom breakage during a suspected fertile period." The second part contained information on the demographic characteristics of the study participants. The third part assessed the sexual and reproductive health knowledge of students; the fourth part evaluated their knowledge, attitude and practice of emergency contraceptive pills while the fifth part was concerned with condom utilization behaviours.

The questionnaire was initially designed taking into consideration similar surveys that have been carried out in other countries [14]. We modified the original questionnaire to suit our context after pre-testing. The questionnaire was distributed to 20 students who did not participate in the study. The completed questionnaires were then studied to identify major issues that needed amendment. As a result, some questions were rephrased.

With the help of assistants from each faculty, the selected students were moved to big classrooms enough to allow free space for privacy and general information about the purpose of the study, importance of their participation, verbal consent, and voluntariness of participation and issues of confidentiality. Data collectors were degree holders who are not known by students. Data quality was maintained through instrument pre-testing, adequate training of data collectors, checking of completed questionnaires and daily supervision by the investigators.

### Data processing and statistical analysis

Data were entered, coded and analysed using SPSS version 17.0 software for windows. Descriptive statistical analysis was used to describe socio-demographic, sexual and reproductive health characteristics, knowledge and attitude of participants and level of use of EOCs and condoms. Binary logistic regression analysis was used to determine association and control for confounding. A p-value less than 0.05 considered as statistically significant.

### Ethical considerations

Ethical approval was obtained from Institutional Review Board of the University of Gondar and informed consent was obtained from each study participant. Letters of permission were obtained from the respective University officials. The questionnaire was anonymous and no names of students were used. The data collected from each student were kept confidential. Any student had the right to withdraw at any point during data collection. Information about safer sex practice was given by health professionals after each data collection session.

## Results

### Socio-demographic characteristics of respondents

A total of 623 students (400 from University of Gondar and 223 from Bahir Dar University) were studied making the response rate of 94.4%. As it is shown in Table 1, most of the respondents 512 (82.2%) were within age group of 20–24 years. The mean (±SD) age of respondents was 20.6(±1.13) years. The majority, 515 (82.7%), were followers of Orthodox Christianity followed by Muslims who account for 50 (8%). Majority 590 (92.7%) of students were unmarried and 132 (21.2%) were originally from rural area (Table 1).

**Table 1:** Socio-demographic characteristics of female students in Bahir Dar and Gondar Universities, Northwest Ethiopia, May 2011

**Characteristic**	**Number**	**Percent**
**Age of Respondent**		
15-19	110	17.7
20-24	512	82.2
≥ 25	1	0.2
**Faculty**		
Health Sciences	193	31.0
VET, Agric, Educ, Engineering	132	21.2
FNCS	49	7.9
FBE,FSSH	249	40.0
**Year of Study**		
Year 2	296	47.5
Year 3	210	33.7
Year 4 And Above	117	18.8
**Ethnicity**		
Amhara	425	68.2
Oromo	64	10.3
Gurage	41	6.6
Tigre	60	9.6
Other	33	5.3
**Religion**		
Orthodox	515	82.7
Muslim	50	8.0
Protestant	47	7.5
Other	11	1.7
**Marital Status**		
Never Married	590	94.7
Married	26	4.2
Others	7	1.1
**Current Residence**		
In campus	556	89.2
Outside Campus	67	10.8
**Residence Of Origin**		
Addis Ababa^a^	198	31.8
Regional Capitals	125	20.1
Woreda Town	168	27.0
Rural Area	132	21.2

^a^Capital city of Ethiopia.

### Sexual and reproductive health characteristics

Among the total female students who participated in this study, 103 (16.5%) responded that have ever had sexual intercourse of which 63 (61.1%) had sex before the age of 20 years and 20 (19.4%) reported that their first partner was a casual sex partner. The mean (±SD) age at first sex was 18.85 (±1.53) years and the minimum age at first was 12 years. Thirty nine (37.9%) have had sex with two or more partners and all students who reported history of unintended pregnancy were from this subset. Forty (38.5%) students reported previous history of sexually transmitted infection (STI). Unintended pregnancy and abortions were observed in 17 (16.5%) and 16 (15.5%), respectively (Table 2).

**Table 2:** Sexual and reproductive health characteristics of female students in Bahir Dar and Gondar Universities, Northwest Ethiopia, May 2011

**Reproductive health characteristic**	**Frequency**	**Percent**
**Ever had sexual intercourse**		
Yes	104	16.7
No	519	83.3
**Age at first sexual intercourse**		
</= 17	16	15.4
18-19	47	45.2
>/= 20	41	39.4
**Person with whom there was first sexual intercourse**		
Steady friend	45	43.3
Casual friend	21	20.2
Husband	23	22.1
Other	15	14.4
**Reason for sexual intercourse**		
Fall in love	56	54.4
Had desire	11	10.7
I got married	19	18.4
Raped	8	7.8
To get money and gifts	6	5.8
Peer pressure and others	4	3.8
**Number of persons student had sexual intercourse with**		
One	65	62.5
Two	18	17.3
Three	6	5.8
Four or more	15	14.4
**History of STI**		
Yes	40	38.5
No	64	61.5
**Unintended pregnancy**		
Yes	17	16.3
No	87	83.7
**History of abortion**		
Yes	16	15.4
No	88	84.6
**Number of Abortions**		
One	10	62.5
Two	6	37.5
**Reasons for abortion**		
Fear of my family	4	25.1
Continue my education	7	43.8
It was unplanned	5	31.3
Economic problem	1	6.3

### Knowledge about contraception

Regarding the respondents knowledge of EOC, 418 (67.1%) ever heard about EOC while 511 (82.0%) had ever heard about condoms as a method of contraception. Two hundred and ninety three (70.1%) correctly identified the recommended 72 hours as the time limit with in which EOC pills must be taken. One hundred and ninety one (45.7%) of participants said that EOC is highly effective in preventing unwanted pregnancy where as 49 (11.7%) and 178 (42.75%) said not effective and didn’t know effectiveness, respectively (Table 3).

**Table 3:** Knowledge of contraceptive methods among female students in Bahir Dar and Gondar Universities, Northwest Ethiopia, May 2011

**Knowledge of Contraception**	**Frequency**	**Percent**
**Pills**		
Yes	483	77.5
No	140	22.5
**IUCD**		
Yes	422	67.7
No	201	32.3
**Injectables**		
Yes	467	75.0
No	156	25.0
**Implants**		
Yes	441	70.8
No	182	29.2
**Knows Condom use**		
Yes	511	82.0
No	112	18.0
**Heard about EOC**		
Yes	418	67.1
No	205	32.9
**Learned about EOC in past**		
Yes	235	47.7
No	258	52.3
**Proper time to take EOC**		
Within 72 hours	293	70.1
Within 120hours/5days/	23	5.5
I don't know	105	25.1
**Effectiveness of EOC to prevent pregnancy**		
Highly effective	191	45.7
Not effective	49	11.7
I don’t know	178	42.5
**EC can prevents STI**		
Yes	243	39
No	380	61
**Condom prevents STI**		
Yes	309	60.5
No	202	39.5
**Ever use of Condom**		
Yes	50	48.1
No	54	51.9

### Attitude and practice towards EOC

Three hundred and sixty nine (59.2%) students were willing to use EOC whenever needed. Among those not willing to use EOC, the reasons were religious prohibition (44.3%), to give birth (25.7%), fear of misinterpretation and rumours (21.0%). More than half, 414(66.5%), of the respondents declared that they will recommend EOC to others and 243 (39%) of respondents think that EOC can prevent STI (Table 4).

**Table 4:** Attitude and utilization of emergency contraception among female students in Bahir Dar and Gondar Universities, Northwest Ethiopia, May 2011

**Characters**	**Number**	**Percent**
**EOC must be available**		
Yes	289	46.4
No	334	53.6
**Willingness to use**		
Yes	369	59.2
No	254	40.8
**Reasons for not using EOC**		
Fear of rumours, misinterpretation	51	21.0
Religious prohibition	112	44.3
I prefer to give birth	65	25.7
Other	29	11.5
**Recommend EOC to others**		
Yes	414	66.5
No	209	33.5
**Think unintended sex is problem of youth today**		
Yes	346	55.5
No	204	32.7
I don't know	73	11.7
**Think unintended pregnancy is problem of youth today**		
Yes	464	74.5
No	98	15.7
I don't know	61	9.8
**Ever used EOC**		
Yes	49	47.1
No	55	52.9

### Factors associated with condom utilization

Table 5 describes the factors associated with condom use. Students who thought that emergency contraceptive is effective in preventing pregnancy were 4 times less likely to use condoms as compared to those who believed that it is not effective (AOR = 0.22 95% CI 0.06, 0.87). on the other hand, female students who believed that condom prevents STI were more than 10 times more likely to use condoms than their counter parts who didn’t believe it prevents STI (AOR = 10.37, 95% CI 1.73, 62.24). Students in the teen age group of <20 years were also 11 times more likely to use condoms than students 20 years old and above (AOR = 11.68 95% CI 1.25, 109.19). The residence knowledge of some method to prevent pregnancy was not significantly associated with condom use (Table 5).

**Table 5:** Factors associated with condom use among female students in Bahir Dar and Gondar Universities, Northwest Ethiopia, May 2011

**Characters**	**Condom Use**		**Crude Odds Ratio (95% C.I.)**	**Adjusted Odds Ratio (95% C.I.)**	**P - value**
	**Yes**	**No**			
**Emergency contraceptive prevents pregnancy**					
Yes	6	17	0.29 (0.08, 0.95)	0.22 (0.06, 0.87)	.030
No	28	23	1	1	
**Current Residence**					
Inside campus	44	44	1.67 (0.5, 5.72)	3.56 (0.56, 22.72)	.179
Outside campus	6	10	1	1	
**Know condom use**					
Yes	43	47	0.91 (0.26, 3.21)	0.073 (0.002, 3.49)	.185
No	7	7	1	1	
**Knows one method to Prevent pregnancy**					
Yes	38	35	1.72 (0.67, 4.43)	2.13 (0.28, 16.19)	.465
No	12	19	1	1	
**Condom prevents STI**					
Yes	40	29	3.45 (1.33, 9.12)	10.37 (1.73, 62.24)	.011
No	10	25	1	1	
**Age group**					
< 20	8	4	2.38 (0.59, 10.22)	11.68 (1.25, 109.19)	.031
≥ 20	42	50	1	1	
**Unwanted pregnancy is youths’ problem**					
Yes	33	37	0.89 (0.39, 2.03)	1.02 (0.26, 4.04)	.977
No	17	17	1	1	

## Discussion

Due to unavoidable administrative reasons, the number of students included in Bahir Dar University was smaller than the allocated size. The majority of our students were in the age group of 20-24 years, the age group considered as sexually active group [15].

According to this study, about 103 (16.5%) have ever had sexual intercourse. This figure is less than other similar studies done in Addis Ababa, Kenya and Ghana reported sexual intercourse in (19.5%) [16], (47.6%) [17] and (38%) [18], respectively, among university female students. This could be due to unwillingness to report having sex with a particular partner or being coerced to have sex.

Nearly two fifth (37.9%) of the students had two or more sexual partners in the past. This figure is much higher than the Ethiopian demographic and health survey results [19]. This might be due to the difference in population that we have used institution based population away from family and are exposed to have more partners than the general population. In addition, university students are at a stage in their lives characterized by searching, discovery, and experimentation, including sexual experimentation [20]. They live and socialise with large numbers of other young adults, which encourages sexual activities that are not mutually monogamous. For this reason, university students are reportedly engaging in unsafe sex, which places them at higher risk than the general public to contract STIs, including HIV and AIDS, as well as unwanted pregnancies [20].

In contrast to rates of unintended pregnancy and induced abortion, the reported lifetime prevalence of STIs was lower for female students than a previous study in South Africa (25%) [21]. This may be due to under-reporting, to the presence of certain STIs such as chlamydia and gonorrhoeal infections that remain largely asymptomatic especially among women [22,23], to embarrassment or financial costs preventing students from seeking medical care, to the limited availability of testing for chlamydia infections in the study area, or to the fact that the university students' sexual network was not developed enough to allow the spread of STI.

Although the unintended pregnancy rate of 16.5% in this study is comparable with Ethiopian Demographic and Health Survey 2005, of 16.2% [24]. Limited condom use and especially multi-partnership are to blame, as evidenced by the fact that all the students who reported history of unintended pregnancy also reported sexual intercourse with multiple sexual partners. One of the most unfavourable outcomes of unplanned, unprotected sex is unintended pregnancy. Unlike in the past when pre-marital pregnancy used to result in compulsory marriage of the girl to the father of her child, today, due to western civilization and the erosion of traditional family and community values, young women are having greater freedom regarding their sexuality. The high cost of education and the desire to continue schooling for enhanced socio-economic status are also pressurizing young women to delay childbearing by resorting to induced abortion as the major method of resolving unwanted pregnancy [25]. In this study, abortion rate is higher than the national average of 2.3% [26-28]. The high abortion rates in these areas are likely the result of many factors, including that the availability of private health care providers draws women from surrounding areas. Previous studies indicated that women seeking induced abortion had a mean age of 23, and the majorities (54%) were single [26-28] which is in agreement with the demographic profile of this study. From the total students reported previous history of abortion, 37.5% reported two or more abortions, highlighting the importance of introducing safe sex education.

The result from this study revealed that more than two third of the respondents had heard of EOC. This is higher than the previous reports from different university students of Ethiopia; 43.5% in Addis Ababa [16], 41.9% in Jimma [29] and 47.6% in Haramaya [30]. Other studies among female students have indicated that 45.1, 56.5, 58 and 61 percent had heard about EOC at university of Uganda, South Africa, Benin and three tertiary institutions in Eastern Nigeria, respectively [31-34]. The result from this study has also revealed that knowledge of correct timing was better than all above studies. This could be due to the high health promotion and availability of EOC in pharmacies. However, more than half of respondents said EOC is not effective and didn’t know about its effectiveness in preventing unwanted pregnancy. Advocacy on EOC use as a back-up contraceptive method for condom failure or non-use should be considered.

In our study, we have identified that more than half of students were willing to use and recommend EOC to others. In addition, female youth who think EOCs are highly effective in preventing pregnancy are nearly four times less likely to use condoms as compared to those who say EOCs are not effective. The findings of this survey support previous studies showing consistent EOC users do not necessarily consider using a condom for STI prevention [6,7].

Despite the potentially devastating outcomes that can result from unprotected sexual intercourse, university students do not consistently take the necessary precautions to protect themselves from contracting an STI and instead see pregnancy prevention as the primary issue. For example, in a survey of 797 college students, 28% cited pregnancy prevention only as the reason for using contraception [35]. This may due to incorrect believe that emergency contraceptive methods provide protection from disease [36]. These findings may be attributed to a lack of knowledge or incorrect beliefs about the effectiveness of oral contraceptives in preventing STIs [35]. Many university students believe that condoms and oral contraceptives are equal choices, rather than understanding that both are required for maximal protection against STIs and unwanted pregnancy [37]. To develop effective disease-prevention messages, better understanding is needed of why women at risk for HIV infection who are using contraceptive methods other than condoms do not use condoms for disease prevention.

Although not statistically associated with condom use, two fifth of respondents think that EOC can prevent STI. This may be due to incorrect beliefs about the effectiveness of oral contraceptives in preventing STIs [35]. According to this study, about 75% of students responded that unintended pregnancy is the problem of youth while only 55% responded that unintended sex is problem. There is some concern that Ethiopian students may be putting themselves at unnecessary risk of STI by choosing the oral contraceptive pill for prevention of pregnancy while remaining at risk of acquiring an STIs through unprotected sex. The problem appears to be with the interplay between the need to prevent pregnancy and the need to protect against STIs. If this trend continues together with the increase in early sex initiation associated with more non-regular and more multiple partnerships, the vulnerable subpopulation of students engaging in unsafe sexual practices will expand, potentially leading to an increased incidence of induced abortion and probably increased future STIs and HIV infection. This concern is supported by many previous studies indicating that an early age of sexual debut is associated with negative outcomes such as unwanted pregnancy, induced abortion, and STIs [38-40].

On the other hand, the level of knowledge on condoms is better than that for the EOC in this study. Participants who believe that condom prevents STIs were more than ten times more likely to use condoms as compared to those who did not believe in condoms. However, sexually active students aged 20 or older and those who thought that EOC is effective in preventing pregnancy are less likely to use condom. This tells, on the other hand, that youth are afraid of the pregnancy than the STIs. In line with this, a study in Canada asked respondents which of AIDS, STIS and pregnancy they worried about most as a possible outcome of sexual intercourse. Pregnancy was selected most often by all groups of students [41]. Another study in Norway showed that the majority of adolescents who use contraception (pills or condoms) do so for protection against unintended pregnancy and not for protection against STIs [6]. Some of the reasons for not using condoms or inconsistently using condoms among students with good knowledge are condom discomfort, possibility of breakage, cost, interruption of sexual activity, need for proper technique, loss of penile sensation, preference for other forms of birth control, or stigma of using a method associated with promiscuity and STIs [42-44]. College women in particular may not use condoms because they are less likely to perceive themselves at risk and believe that condoms do not play a role in a relationship based on love, trust, and commitment [14,45,46].

### Limitations

This study has some limitations. First, the study was carried out in only two universities of Northwest Ethiopia, and thus the finding cannot be generalized to EOC users in Ethiopia. Second, we cannot guarantee that students provided honest answers to the questions, since the survey involved a sensitive matter (i.e. sex), therefore; it is important to remember that the reliability of results of this study are dependent upon the accuracy of the responses. Its cross-sectional design was also limited in evaluating cause-and-effect associations. In addition, the study is limited to women. Knowledge and attitudes of men need to be considered as well if we hope to make changes in the use of contraceptives in this population. However, to our knowledge this article is the first of its type to look at effect of emergency oral contraception on condom use among students in Ethiopia.

## Conclusions

This study showed that the awareness of emergency contraception among the female students was high. The students had also good knowledge of the timeframe for the use of EOC. However, students aged 20 or older and those who have positive attitude towards the effectiveness of EOC to prevent unwanted pregnancy are less likely to use condom. Therefore, women at risk for STIs who are not using condoms for pregnancy prevention may not use condoms for prevention of HIV infection and other STIs. The preference for the pill may make teenagers less prepared to practice STI protective behaviours in specific situations. Therefore, there is an urgent need to educate young people in universities about reproductive health and family planning and skills on how to prevent HIV/STIs including unwanted pregnancy. There is also the need to include reproductive health in the school curriculum.

## Abbreviations

AOR: (Adjusted odds ratio); EOC: (Emergency oral contraception); HIV: (Human immunodeficiency virus); AIDS: (Acquired immunodeficiency syndrome); STIs: (Sexually transmitted infections); OR: (Odds ratio); SPSS: (Statistical package for social science).

## Competing interests

The authors declare that they have no competing interests.

## Authors' contributions

BW: study design, data collection and analysis, interpret the data, draft and reviewed the manuscript; YB: data collection, Data entry, data analysis and reviewed the manuscript; BM: data collection, Data entry, data analysis and reviewed the manuscript; BA: conception of the research idea, design of the study, carrying out the data collection, and drafting the manuscript. All authors have read and approved of the final version of the manuscript.
